# The immunomodulating V and W proteins of Nipah virus determine disease course

**DOI:** 10.1038/ncomms8483

**Published:** 2015-06-24

**Authors:** Benjamin A. Satterfield, Robert W. Cross, Karla A. Fenton, Krystle N. Agans, Christopher F. Basler, Thomas W. Geisbert, Chad E. Mire

**Affiliations:** 1 Galveston National Laboratory, University of Texas Medical Branch, 301 University Boulevard, Galveston, Texas 77555, USA; 2 Department of Microbiology and Immunology, University of Texas Medical Branch, 301 University Boulevard, Galveston, Texas 77555, USA; 3 Department of Microbiology, Icahn School of Medicine at Mount Sinai, New York, New York 10029, USA

## Abstract

The viral determinants that contribute to Nipah virus (NiV)-mediated disease are poorly understood compared with other paramyxoviruses. Here we use recombinant NiVs (rNiVs) to examine the contributions of the NiV V and W proteins to NiV pathogenesis in a ferret model. We show that a V-deficient rNiV is susceptible to the innate immune response *in vitro* and behaves as a replicating non-lethal virus *in vivo*. Remarkably, rNiV lacking W expression results in a delayed and altered disease course with decreased respiratory disease and increased terminal neurological disease associated with altered *in vitro* inflammatory cytokine production. This study confirms the V protein as the major determinant of pathogenesis, also being the first *in vivo* study to show that the W protein modulates the inflammatory host immune response in a manner that determines the disease course.

## Abstract (web summary)

Nipah virus (NiV) can be transmitted from bats and other animals to humans, causing severe encephalitis and respiratory disease. Here, Satterfield *et al.* show that the W protein of NiV modulates the host immune response and determines disease course in a ferret model of infection.

## Graphical abstract

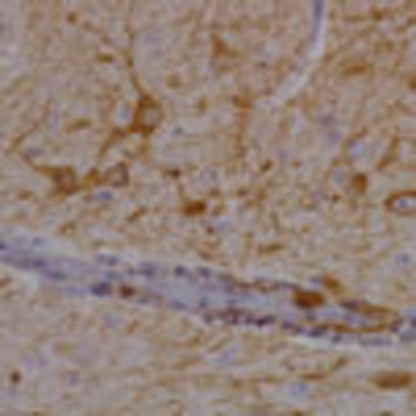

Nipah virus (NiV) belongs to the family *Paramyxoviridae* and it emerged recently as a human pathogen causing acute respiratory distress and severe encephalitis with systemic vasculitis^1^,^2^. Pulmonary or encephalitic NiV-mediated disease can be lethal with case fatality rates ranging from 38 to 92% (refs 3, 4); many of the survivors experience long-term neurological sequelae^5^. Since the initial outbreak of NiV in 1998–1999 in Malaysia and Singapore^6^, outbreaks have occurred almost every year in Bangladesh and northeastern India^7^,^8^,^9^,^10^,^11^,^12^ and with a recent outbreak occurring in the Philippines^13^. Research with infectious NiV is restricted to Biosafety Level 4 (BSL-4) laboratories; therefore, much is still unknown about NiV pathogenesis^14^,^15^ including what viral determinates contribute to the pulmonary and encephalitic components of NiV disease.

The NiV P gene encodes not only the P protein but three additional proteins, namely C, W and V^16^. The C protein utilizes an alternative start codon and shares no homology with P^16^, while the V and W proteins are produced through mRNA editing^17^,^18^. This results in P, W and V proteins that share a common N-terminal domain (upstream of the editing site) with each having a unique C-terminal domain (downstream of the editing site).

NiV can naturally infect bats, humans, pigs, dogs, cats and other species^19^,^20^,^21^. Current experimental animal models for NiV infection include hamsters^22^, cats^23^,^24^, pigs^24^,^25^, ferrets^26^, squirrel monkeys^27^ and African green monkeys^28^. Recently, interferon (IFN) receptor knockout (ko) mouse^29^ and human lung xenograph mouse^30^ models have been established, although non-modified mice only develop a mild, self-limited pulmonary infection^31^. Each model has certain advantages and disadvantages; among the small and medium-sized mammals, hamsters can develop meningoencephalitis (low dose) or respiratory disease (high dose)^32^. Acute respiratory disease occurs in the cat model^23^; however, the recently developed ferret model of NiV infection demonstrates both respiratory and neurological disease as well as systemic vasculitis^26^,^33^, thus accurately modelling all major aspects of NiV-mediated disease in humans^2^.

The IFN response through IFN-α and -β plays a critical role in controlling viral infections by signalling both infected and non-infected cells to enter into an antiviral state. This signalling occurs through the Janus Kinase (JAK)/Signal Transducer and Activator of Transcription (STAT) pathway. Many viruses have evolved means of inhibiting IFN signalling^34^. Paramyxoviruses have evolved mechanisms whereby the P gene products (P, V, W and C) inhibit the JAK/STAT signalling pathway^35^,^36^ through a common N-terminal domain that can bind to STAT1 and inhibit its phosphorylation^37^. The NiV V protein also inhibits the antiviral functions of RIG-I (ref. 38) and MDA5 (ref. 39) through phosphatase PP1 (ref. 40), and STAT2 (ref. 41) through direct sequestration. The NiV V and W proteins have also been shown to suppress expression of the IFN-β and IFN-stimulated gene 54 (ISG54) promoters in HEK 293T cells, with the W protein appearing the most potent^42^. The W protein potentially destabilizes IFN regulatory transcription factor 3, thereby blocking both Toll-like receptor 3 and Inhibitor of κB kinase ε (IKKε) signalling pathways, whereas the V protein only blocks IKKε signalling^42^. Therefore, it has been suggested that both the V and W proteins play important roles in allowing NiV to evade the innate immune system and establish systemic infection with W likely playing a more prominent role^43^. However, it is unclear whether the W protein is able to function similarly in all cell types since evidence suggests that it is primarily found in the nucleus in some cell types^42^,^44^ but primarily in the cytoplasm of endothelial cells^44^. Much of the data are a result of overexpression studies and do not always align with infection-based studies^45^.

To investigate the role of the V and W proteins in NiV infection, recombinant NiV (rNiV) strains that lack the ability to express each of these proteins have been examined *in vitro*^46^ and in the hamster model^47^. However, the backbone used in the creation of the various rNiV-Malaysia (rNiV_M_) constructs to create the recombinant wild-type virus appeared attenuated *in vivo* when compared with the parental virus^47^,^48^, possibly because of additional restriction sites introduced for cloning. This attenuation makes the *in vivo* data difficult to interpret.

Here we utilize reverse genetics to produce rNiV_M_ strains with no additional restriction sites in either a wild-type (rNiV_M_-wt) genotype or a knocked out genotype for the W protein (rNiV_M_-W^ko^) or V protein (rNiV_M_-V^ko^). These rNiVs are characterized in primary cells *in vitro* followed by studies in the ferret model in order to determine what role the V and W proteins play in the pathogenesis of NiV-mediated disease. Our results suggest that the V protein is the major determinate of pathogenesis and lethality, while the W protein contributes to the control of the inflammatory response, with rNiV_M_-W^ko^ altering the disease course from the pulmonary tissues to the neural tissues without altering disease lethality. To our knowledge, this is the first report to show that ko of a paramyxovirus innate immune-modulatory protein, the NiV W protein, can alter disease course but not alter virulence *in vivo*.

## Results

### rNiV_M_ recovery and *in vitro* characterization

**Figure 1:**
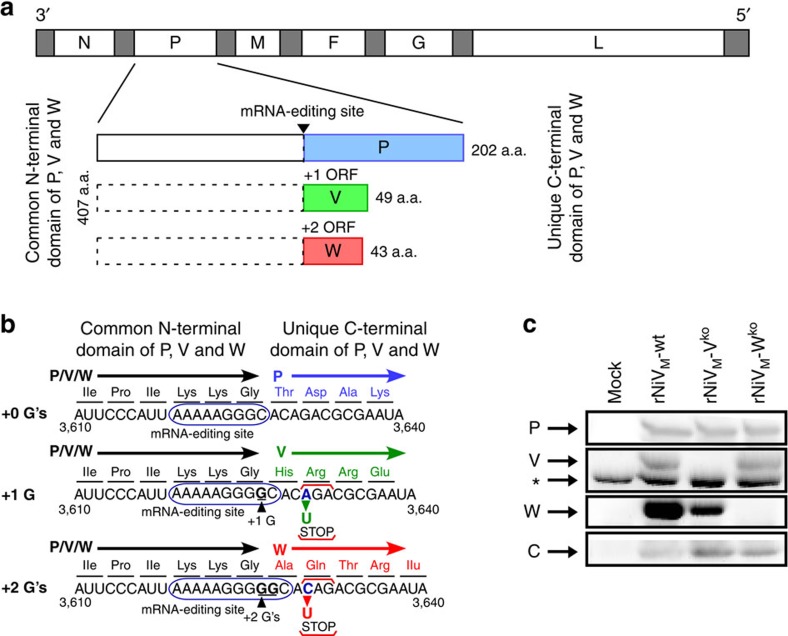
rNiV_M_ genome design. (**a**) Schematic of the rNiV_M_ genome with the name of each gene indicated (N, P, M, F, G and L). White segments indicate open reading frames (ORFs) and grey segments represent noncoding regions of the genome. Insert magnifies the P gene, indicates the mRNA-editing site that allows for the production of the W and V proteins as well as the P protein from the P gene, shows the lengths of the common N-terminal domain and unique C-terminal domains and indicates the relative position of the ORFs of V and W after the mRNA-editing site. (**b**) The nucleotide and amino-acid sequences of the P, V and W ORFs around the mRNA-editing site are shown. Nucleotide changes, indicated by green and red arrows and bold letters, were incorporated into the *P* gene to introduce early stop codons in the V and W ORFs shortly after the mRNA-editing site (blue oval) to generate the rNiV_M_-V^ko^ and rNiV_M_-W^ko^ mutants, respectively. Numbers flanking the nucleotide sequences indicate positive-sense antigenomic position. (**c**) Western blot analysis of Vero cell lysates either mock-infected, or infected with rNiV_M_-wt, rNiV_M_-V^ko^ or rNiV_M_-W^ko^. NiV P-, V-, W- and C-specific polyclonal antibodies were used to detect the presence or absence of the respective proteins. * nonspecific binding of the V polyclonal antibody. Full blots can be seen in Supplementary Fig. 1.

Through reverse genetics, rNiV_M_-wt, rNiV_M_-W^ko^ and rNiV_M_-V^ko^ strains were successfully recovered using previous methods^43^ (Fig. 1a,b). Western blot analysis of infected Vero cell lysates demonstrated that the W and V proteins were not being expressed by rNiV_M_-W^ko^ and rNiV_M_-V^ko^, respectively (Fig. 1c). In addition, we noted increased production of C protein after removing W or V protein expression as has been noted elsewhere^46^.

**Figure 2:**
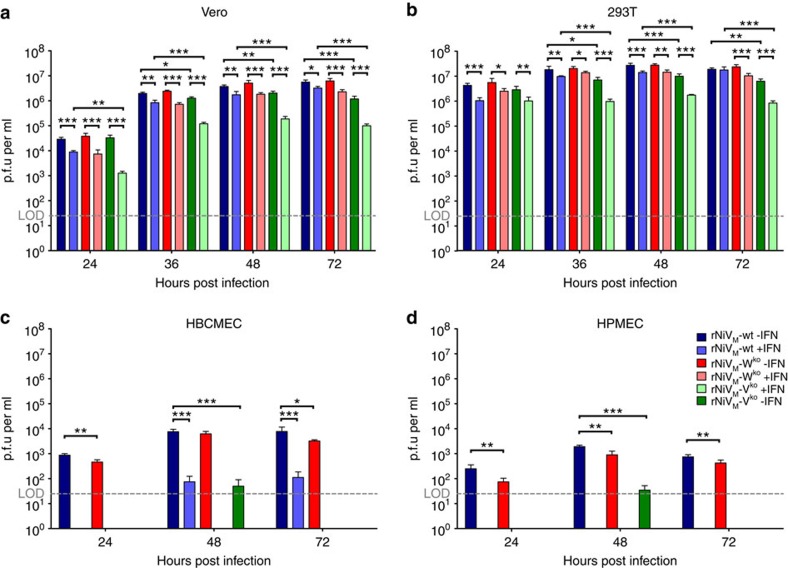
rNiV_M_ *in vitro* growth kinetics. Growth kinetics in Vero cells (**a**), 293T cells (**b**), HBCMECs (**c**) and HPMECs (**d**) either with or without 12-h pre-treatment of IFN-α. Blue bars indicate rNiV_M_-wt, red bars indicate rNiV_M_-W^ko^ and green bars indicate rNiV_M_-V^ko^. Dark colours indicate no pre-treatment with IFN-α and light colours indicate 12-h pretreatment with IFN-α. Error bars show s.d. LOD: limit of detection of 25 p.f.u. per ml. Analysis of variance (ANOVA) with Dunnett’s multiple comparison test; *N*=4. *P value<0.05; **P value<0.01; ***P value<0.001 compared with non-IFN-treated cells infected with the same rNiV_M_ strain, or rNiV_M_-wt either with or without IFN treatment as indicated.

Multicycle growth kinetics were determined for each rNiV_M_ strain in Vero (Fig. 2a) and HEK 293T cells (Fig. 2b). In both cell lines the growth curves and peak titres were almost identical for the rNiV_M_-wt and rNiV_M_-W^ko^ strains, while the rNiV_M_-V^ko^ strain grew to moderately lower titres (0.25–0.5 logs) beginning at 36 h post infection (p.i.). The Vero cell data observed here appear to be closer to the rNiV_M_-wt than previously reported^46^,^47^. To examine whether the W and V proteins were important in the context of virus infection for overcoming the innate immune response, multicycle growth kinetics were assessed in Vero and HEK 293T cells pre-stimulated with 1,000 U ml^−1^ of Universal IFN-α for 12 h before infection. The rNiV_M_-W^ko^ strain again grew to similar titres as the rNiV_M_-wt strain, although both strains grew to moderately lower titres (0.25–0.5 logs) than in cells not pre-treated with IFN-α. The rNiV_M_-V^ko^ strain, however, grew to markedly lower titres (1.0–1.75 logs) as compared with cells not pre-treated with IFN-α.

### rNiV_M_-infected primary human endothelial cells

Endothelial cells in the brain and lungs are common targets for NiV infection^2^; therefore, primary human brain and lung microvascular endothelial cells were infected with the rNiV_M_ strains to determine the multicycle growth kinetics. In human brain cerebrum microvascular endothelial cells (HBCMEC) rNiV_M_-W^ko^ had similar kinetics to rNiV_M_-wt (Fig. 2c), but grew to moderately lower titres (0.5 logs) in human pulmonary microvascular endothelial cells (Fig. 2d).

To assess the effect of the innate immune response on multicycle growth kinetics in primary endothelial cells, HBCMECs and HPMECs were pre-treated with 1,000 U ml^−1^ of Universal IFN-α for 12 h before infection. IFN-α was very potent at blocking rNiV_M_ production in both HPMECs and HBCMECs; compared with untreated cells, cells pre-treated with IFN-α produced low levels of rNiV_M_-wt (2 logs lower) at 48 and 72 h p.i. in HPMECs, while no rNiV_M_-wt production was detected in HBCMECs pre-treated with IFN-α at any time points. No virus was detectable in either IFN-α pre-treated HBCMECs or HPMECs infected with either rNiV_M_-W^ko^ or rNiV_M_-V^ko^.

**Figure 3:**
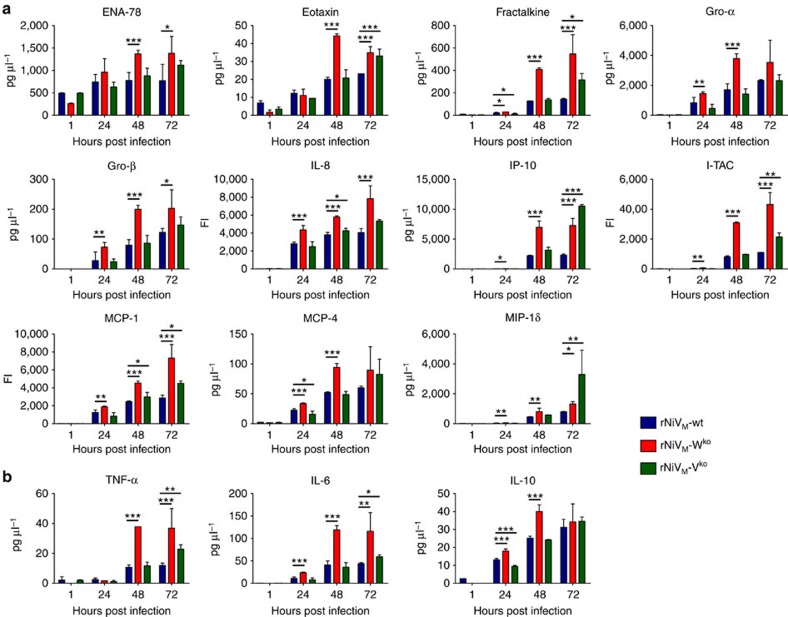
Selected chemokine/cytokine levels in supernatants of infected HPMECs. Inflammatory chemokine levels (**a**) and innate immune cytokine levels (**b**) of HPMECs infected with rNiV_M_-wt (blue), rNiV_M_-W^ko^ (red) or rNiV_M_-V^ko^ (green). Error bars show s.d. ANOVA with Dunnett’s multiple comparison test; *N*=4. *P value<0.05; **P value<0.01; ***P value<0.001 compared with rNiV_M_-wt, FI, fluorescence intensity.

To examine the chemokine/cytokine response after initial target cell infection, supernatants from the infected HPMECs were used to quantify the levels of 42 chemokines/cytokines using Bio-Plex or ELISA (Supplementary Table 1). Interestingly, many of the pro-inflammatory and leukocyte-attracting chemokines including ENA-78, Eotaxin, Fractalkine, Gro-α, Gro-β, IL-8, IP-10, I-TAC, MCP-1, MCP-4 and MIP-1δ (Fig. 3a), and innate immune cytokines including TNF-α, IL-6 and IL-10 (Fig. 3b) were observed at higher levels in supernatants from HPMECs infected with rNiV_M_-W^ko^ than in HPMECs infected with either rNiV_M_-wt or rNiV_M_-V^ko^.

### Clinical disease in ferrets

**Figure 4:**
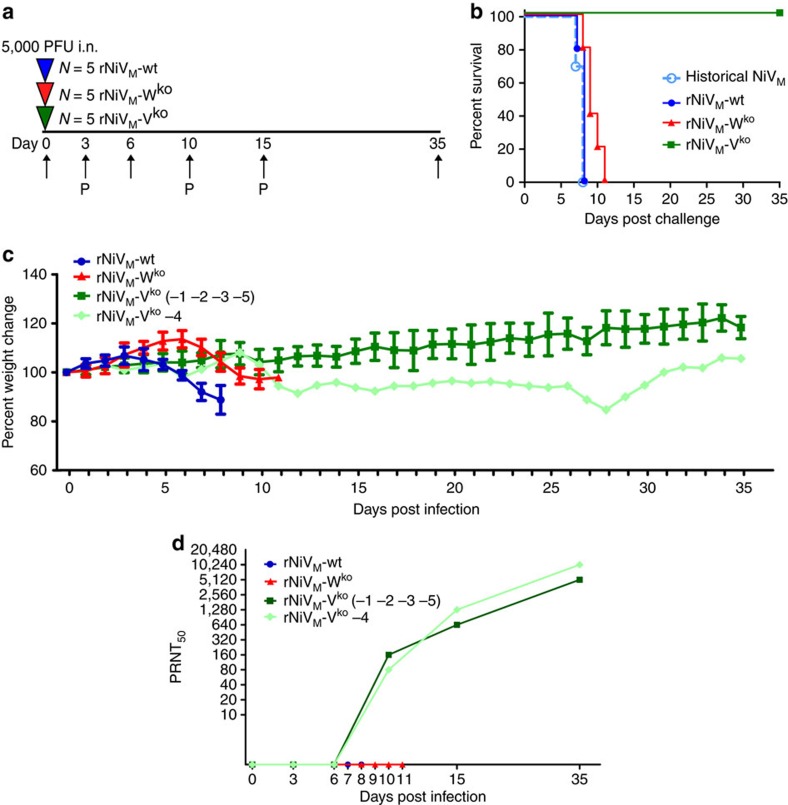
Clinical disease in ferrets after experimental infection with rNiV_M_. (**a**) Flow chart showing the day of infection (triangles) and days of sample collection (arrows). P, Pilot animals (numbers -1 and -2 from each cohort) were not sampled on these days. (**b**) Kaplan–Meier survival curve for ferrets infected with rNiV_M_-wt (blue circles), rNiV_M_-W^ko^ (red triangles) and rNiV_M_-V^ko^ (green squares); historical natural, non-recombinant NiV_M_ controls have been included (light blue dotted line, open circles; *N*=10). Log-rank (Mantel–Cox) test; *N*=5 for all rNiV_M_ ferret cohorts. *P value<0.05; **P value<0.01 compared with rNiV_M_-wt. (**c**) Weight change for animals from the rNiV_M_-wt cohort (blue circles), rNiV_M_-W^ko^ cohort (red triangles), rNiV_M_-V^ko^ cohort animals −1, −2, −3 and −5 (dark green squares) and animal rNiV_M_-V^ko^-4 (light green diamonds). Ferret rNiV_M_-V^ko^-4 is separated from the rest of the rNiV_M_-V^ko^ cohort because it showed distinct clinical signs. (**d**) Neutralizing antibody titres for each ferret cohort. Error bars show s.d.

Ferrets are considered an excellent model for studying NiV-mediated disease as they demonstrate respiratory, neurological, vascular and systemic disease similar to that seen in humans infected with NiV^26^,^49^,^50^. Three cohorts of five ferrets each were challenged intranasally (i.n.) with ∼5,000 p.f.u. of rNiV_M_-wt, rNiV_M_-W^ko^ or rNiV_M_-V^ko^. Post challenge, animal weight, temperature and clinical score were assessed daily, with blood drawn on days 3, 6, 10, 15 and 35, or on the day an animal succumbed to disease (Fig. 4a).

All animals in the rNiV_M_-wt cohort succumbed to rNiV_M_-mediated disease on days 7–8 p.i. (Fig. 4b, blue) with fever, severe respiratory disease and moderate neurological signs (Table 1). This is the same time to death as historical control ferrets infected with ∼5,000 p.f.u. of natural, non-recombinant NiV_M_ (Fig. 4b, light blue dotted line). All animals in the rNiV_M_-W^ko^ cohort succumbed to disease on days 8–11 p.i. (Fig. 4b, red) with fever, moderate respiratory disease and severe neurological signs (Table 1). All animals in the rNiV_M_-V^ko^ cohort developed fever but no respiratory signs, and all animals survived the challenge (Fig. 4b, dark green; Table 1). One of the animals, rNiV_M_-V^ko^-4, experienced some weight loss and developed mild neurological signs beginning on day 12 and resolving by day 23 p.i. (Fig. 4b, light green; Table 1). The other animals in the rNiV_M_-V^ko^ cohort did not show any neurological signs at any point in the study.

Additional clinical signs observed in animals from the rNiV_M_-wt and rNiV_M_-W^ko^ cohorts included depression; lethargy; ocular, nasal and oral discharge; sneezing; nasal and oral frothing, dehydration; rales; ataxia; tremors and myoclonus. Some animals in the rNiV_M_-W^ko^ cohort also had periodic seizures (Table 1).

Clinical biochemistry and haematological analysis was performed on all blood samples taken. As the disease progressed, common findings in animals from the rNiV_M_-wt and rNiV_M_-W^ko^ cohorts included thrombocytopaenia, lymphopenia, hypoalbuminemia, >3-fold increase in blood urea nitrogen and hyperglycaemia. These were also observed in a few of the animals from the rNiV_M_-V^ko^ cohort, although not as consistently and to the degree as for the other cohorts (Table 1).

### Neutralizing antibody response

To assess the humoral immune response to infection with all rNiVs, serum samples were measured for the amount of neutralizing antibody using a plaque reduction neutralization assay (PRNT) and reported as the dilution at which plaques were reduced by at least 50% compared with controls (PRNT_50_). No significant neutralizing antibody was detected in any animals from the rNiV_M_-wt and rNiV_M_-W^ko^ cohorts at any time points, while significant levels of neutralizing antibody were detected in all animals from the rNiV_M_-V^ko^ cohort beginning at day 10 p.i. with levels increasing on days 15 and 35 p.i. (Fig. 4D).

### Gross pathology

**Figure 5:**
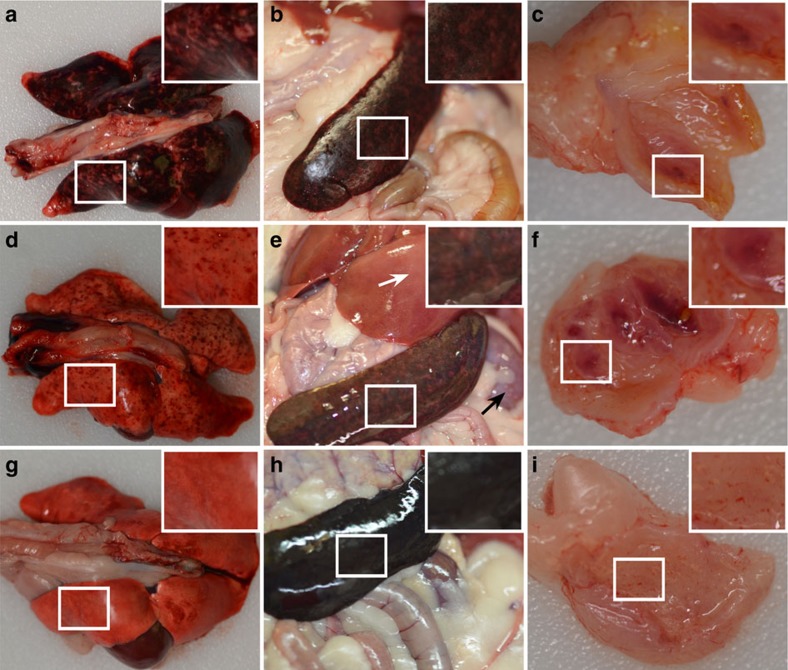
Gross pathology of lungs, spleens and urinary bladders from rNiV_M_-infected ferrets. Representative gross pathology of lungs (**a**,**d**,**g**), spleen (**b**,**e**,**h**) and urinary bladders (**c**,**f**,**i**) taken from ferrets infected with rNiV_M_-wt (**a**–**c**), rNiV_M_-W^ko^ (**d**–**f**) and rNiV_M_-V^ko^ (**g**–**i**). Inserts show magnified regions of each specimen. Multifocal to coalescing haemorrhage and necrosis of all lung lobes is seen in rNiV_M_-wt-infected ferrets (**a**), while much fewer and smaller numbers of haemorrhagic and necrotic foci are seen in rNiV_M_-W^ko^-infected ferrets (**d**). Splenomegaly and multifocal necrosis are seen in spleens from rNiV_M_-wt (**b**)- and rNiV_M_-W^ko^ (**e**)-infected ferrets. Kidneys from ferrets infected with rNiV_M_-W^ko^ showed multifocal haemorrhage and necrosis (**e**, black arrow) and livers were diffusely pale and reticulated (**e**, white arrow). Similar lesions were seen in rNiV_M_-wt-infected ferrets (not shown). Very minimal to no pathology was observed in lungs (**e**) and spleens (**f**) from rNiV_M_-V^ko^-infected ferrets. Urinary bladders showed large haemorrhagic lesions in ferrets infected with rNiV_M_-wt (**c**) and rNiV_M_-W^ko^ (**f**); very small, multifocal haemorrhagic lesions seen in the urinary bladder of ferret rNiV_M_-V^ko^-4 (**i**); no lesions were observed in the urinary bladders of the other ferrets from the rNiV_M_-V^ko^ cohort.

Necropsies were performed on each animal after they succumbed to disease or when being euthanized at the study end point. The gross pathology findings showed a marked difference in the lung lesions between the three cohorts, with all animals (5/5) in the rNiV_M_-wt cohort displaying multifocal to coalescing haemorrhagic and necrotizing pneumonia (Fig. 5a), all animals (5/5) in the rNiV_M_-W^ko^ cohort had multifocal pinpoint haemorrhagic and necrotizing pneumonia (Fig. 5d), although with considerably less severe gross lesions than seen in the rNiV_M_-wt cohort, and the rNiV_M_-V^ko^ cohort that showed no significant gross pulmonary lesions (Fig. 5g). The spleens of all animals (5/5) in the rNiV_M_-wt cohort and all animals (5/5) in the rNiV_M_-W^ko^ cohort were enlarged and mottled with multifocal white and dark red patches indicating splenomegaly and multifocal necrosis, respectively (Fig. 5b,e). In contrast, the spleens of the animals in the rNiV_M_-V^ko^ cohort were uniformly dark with no necrosis (Fig. 5h) and with some (3/5) animals showing minimal splenomegaly. Some animals (2/5) in the rNiV_M_-wt cohort had mucosal haemorrhagic lesions in the urinary bladder (Fig. 5c), all animals (5/5) in the rNiV_M_-W^ko^ cohort had mucosal haemorrhagic lesions in the urinary bladder (Fig. 5f) and only a single animal (1/5) in the rNiV_M_-V^ko^ cohort had multiple pinpoint mucosal reddening of the urinary bladder (Fig. 5i). Notably, this animal was rNiV_M_-V^ko^-4, which was the only animal in this cohort to demonstrate any clinical signs of disease other than fever.

The only obvious gross lesion in the brain of any animal in the rNiV_M_-wt cohort was the presence of a blood clot under the meninges and overlying the frontal lobe of one animal that was found dead. In contrast, all animals (5/5) in the rNiV_M_-W^ko^ cohort had congestion of the meningeal blood vessels with a single animal having a focal pooling of blood under the meninges overlying the right lateral hemisphere. Poorly defined sulci and gyri of the brain, interpreted as oedema was the only gross brain lesion seen in any animal from the rNiV_M_-V^ko^ cohort. Once again, this animal was rNiV_M_-V^ko^-4, which was the only animal in this cohort to demonstrate any clinical neurological signs of disease and have them resolved over the course of the experiment.

### Histopathology and immunohistochemistry

**Figure 6:**
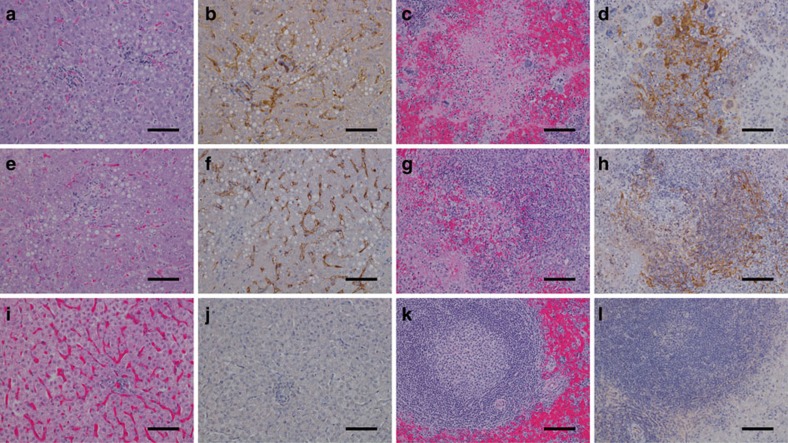
H&E and immunohistochemistry of the ferret liver and spleen. Representative H&E (**a**,**c**,**e**,**g**,**i**,**k**) and immunohistochemistry labelled with a NiV N protein-specific polyclonal rabbit antibody (**b**,**d**,**f**,**h**,**j**,**l**). The liver (**a**,**b**,**e**,**f**,**i**,**j**) and spleen (**c**,**d**,**g**,**h**,**k**,**l**) from representative ferrets infected with rNiV_M_-wt (**a–d**), rNiV_M_-W^ko^ (**e–h**) and rNiV_M_-V^ko^ (**i–l**). Images taken, liver × 20, spleen × 20; scale bar, 100 μm.

Tissues were examined with haematoxylin and eosin (H&E) staining and with immunohistochemistry (IHC) using antibodies specific to the NiV N protein. Hepatic histopathologic lesions for all animals in the rNiV_M_-wt (Fig. 6a) and rNiV_M_-W^ko^ (Fig. 6e) cohorts included minimal to moderate hepatocellular degeneration/necrosis with minimal to mild vacuolar change, moderate congestion with occasional sinusoidal leukocytosis (neutrophilia) and moderate to severe periportal lymphoplasmacytic infiltrates. Strong immunolabelling for NiV antigen was present in all animals in the rNiV_M_-wt (Fig. 6b) and rNiV_M_-W^ko^ (Fig. 6f) cohorts. Immunolabelling for NiV was present in multifocal regions of the sinusoidal lining cells, few scattered mononuclear cells within the sinusoids (Kupffer cells), the endothelium of medium to large caliber vessels and scattered mononuclear inflammatory cells, that were largely centred around areas of necrosis. In animals from the rNiV_M_-V^ko^ (Fig. 6i) cohort, there was no hepatocellular degeneration/necrosis noted, and no immunolabelling for NiV antigen (Fig. 6j). Splenic histopathologic lesions for all animals in the rNiV_M_-wt (Fig. 6c) and rNiV_M_-W^ko^ (Fig. 6g) cohorts included moderate to marked (rNiV_M_-wt) or mild to moderate (rNiV_M_-W^ko^) diffuse lymphoid depletion of lymphoid follicles, syncytial cell formation, haemorrhage and fibrin deposition of the white pulp with accumulation of viable neutrophils, degenerative neutrophils and cellular debris. Strong immunolabelling for NiV antigen was present in all animals in the rNiV_M_-wt (Fig. 6d) and rNiV_M_-W^ko^ (Fig. 6h) cohorts. Immunolabelling for NiV was present in scattered mononuclear cells (largely centred on lymphoid follicles/germinal centre remnants), syncytial cells and scattered endothelium. No splenic histopathologic lesions were noted in animals in the rNiV_M_-V^ko^ cohort (Fig. 6k) and no immunolabelling for NiV antigen was noted (Fig. 6l).

**Figure 7:**
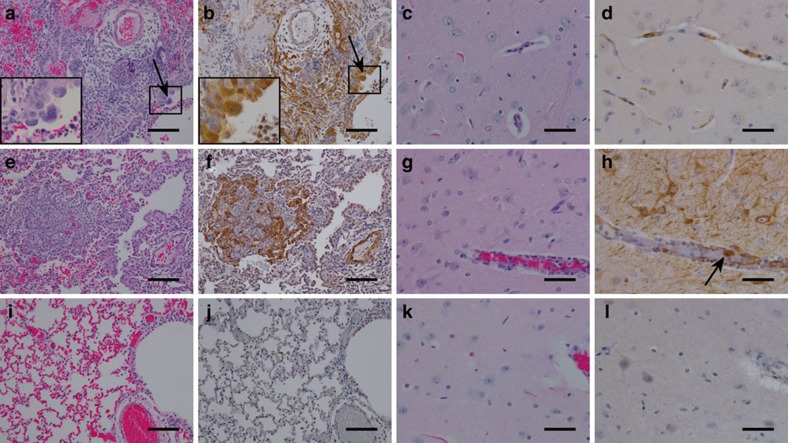
H&E and immunohistochemistry of the ferret lung and brain. Representative H&E (**a**,**c**,**e**,**g**,**i**,**k**) and immunohistochemistry labelled with a NiV N protein-specific polyclonal rabbit antibody (**b**,**d**,**f**,**h**,**j**,**l**). The lung (**a**,**b**,**e**,**f**,**i**,**j**) and brain (**c**,**d**,**g**,**h**,**k**,**l**) from representative ferrets infected with rNiV_M_-wt (**a–d**), rNiV_M_-W^ko^ (**e–h**) and rNiV_M_-V^ko^ (**i–l**). White arrows and magnified inserts indicate syncytial cells (**a**,**b**,**h**) and black arrow indicates neuron with immunolabelling (**h**). Images taken: lung, × 20, brain, × 40; scale bar, 100 μm for the lung and 50 μm for the brain.

Pulmonary histopathologic lesions in all lung lobes of animals in the rNiV_M_-wt (Fig. 7a) cohort and to a lesser extent all animals in the rNiV_M_-W^ko^ (Fig. 7e) cohort included interstitial pneumonia with nodular inflammation and necrosis of the alveolar septae near the terminal bronchioles and occasional, small syncytial cell formation of endothelium and/or respiratory epithelial cells. Strong immunolabelling for NiV antigen in the rNiV_M_-wt (Fig. 7b) and rNiV_M_-W^ko^ (Fig. 7f) cohorts was present in scattered mononuclear, endothelial and respiratory epithelium. Overall, the nodular inflammation and necrosis of the terminal bronchioles appeared more discrete, larger and more haemorrhagic in sections from the rNiV_M_-W^ko^ cohort compared with rNiV_M_-wt; however, there was more diffuse oedema and haemorrhage noted throughout the lung lobes from the rNiV_M_-wt cohort. The rNiV_M_-V^ko^ (Fig. 7i) cohort had rare areas of minimal interstitial pneumonia or oedema; however, no other significant histopathologic lesions were noted and no immunolabelling for NiV antigen was identified (Fig. 7j).

No histopathologic lesions were noted on routine H&E staining of the brain for all animals in the rNiV_M_-wt (Fig. 7c) cohort. However, strong immunolabelling for NiV antigen was present in the endothelium of small caliber vessels within multiple sections of the brain (cerebrum, brainstem, choroid plexus and meninges) in all animal of the rNiV_M_-wt (Fig. 7d) cohort. No histopathologic lesions were noted in the brain of animals in the NiV_M_-W^ko^ (Fig. 7g) cohort; however, congestion of vessels and haemorrhage was noted expanding the meninges. Animals in the rNiV_M_-W^ko^ cohort had multifocal areas with strong immunolabelling for NiV antigen within the endothelium and neurons in the cerebrum, cerebellum/brainstem and/or hippocampus (Fig. 7h). No animals in the rNiV_M_-V^ko^ cohort showed any histologic lesions (Fig. 7k) or immunolabelling for NiV antigen (Fig. 7l) in the brain, including the endothelium.

### Viral load

**Figure 8:**
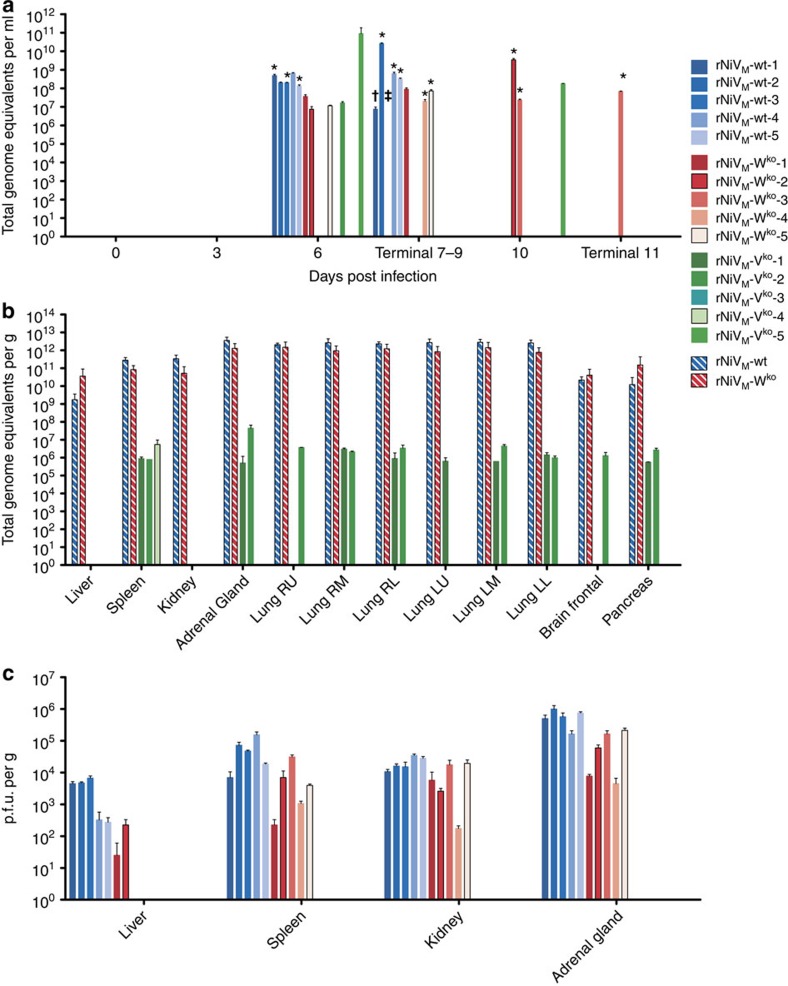
Viral load in rNiV_M_-infected ferrets. Viral load in ferrets as detected by GEq using qRT–PCR from (**a**) whole blood as GEq per ml and from (**b**) tissues as GEq per g (**c**) p.f.u. per g of rNiV_M_ isolated from tissues of ferrets after necropsy. Hashed blue and red lines represent the average of all animals in the rNiV_M_-wt and rNiV_M_-W^ko^ cohorts, respectively. Error bars show s.d. *N*=5 for hashed lines; *N*=2 for all other lines. Right upper (R.U.), right middle (R.M.), right lower (R.L.), left upper (L.U.), left middle (L.M.), left upper (L.U.) *indicates samples in which rNiV_M_ was successfully isolated from whole-blood samples. **†**Animal was found dead, blood was examined using qRT–PCR but no attempt was made to isolate virus. **‡**Animal was found dead and no attempt was made to examine blood using qPCR or to isolate virus.

To assess the viral load in animals, virus isolation and quantitative real-time PCR (qRT–PCR) were attempted with qRT–PCR used to quantify the amount of viral genomes present in whole blood (Fig. 8a). All rNiV_M_-wt cohort animals had detectable levels of viral genome on day 6 p.i. and remained viraemic until they succumbed to disease. Three of the animals in the rNiV_M_-W^ko^ cohort had detectable levels of viral genome in their blood on day 6 p.i., and all animals became viraemic before succumbing to disease. Only two animals in the rNiV_M_-V^ko^ cohort (rNiV_M_-V^ko^-2 and -5) had detectable level of viral genome in whole blood on day 6 p.i. and on day 10 p.i. for rNiV_M_-V^ko^-5.

Viral loads were also measured in 10% weight/volume tissue homogenates taken at necropsy; RNA was extracted and the presence of viral genomes was detected and quantified using qRT–PCR (Fig. 8b). High levels of viral genome were detected in all tissues assayed for all animals in the rNiV_M_-wt and rNiV_M_-W^ko^ cohorts; only three animals from the rNiV_M_-V^ko^ cohort had detectable levels of viral genome in some tissues, and these were considerably lower in value (5–6 logs lower). Virus isolation was attempted from the liver, spleen, kidney and adrenal gland in PCR-positive samples, and virus was isolated from all PCR-positive samples from animals in the rNiV_M_-wt and rNiV_M_-W^ko^ cohorts, with the exception of some of the liver samples in the rNiV_M_-W^ko^ cohort; however, virus was not isolated from any samples of the rNiV_M_-V^ko^ cohort (Fig. 8c).

## Discussion

Previous studies using *in vitro* expression systems of NiV W and V proteins suggested that both proteins were able to antagonize multiple aspects of IFN production and signalling, with the observation that the W protein was the more potent antagonist^37^,^38^,^41^,^42^,^43^. However, one study examined this *in vivo*^47^ and showed that a V^ko^ strain of rNiV_M_, called rNiV(V-) was completely attenuated in hamsters with no hamsters succumbing to disease, while hamsters infected with the W^ko^ strain, termed rNiV(W-), succumbed to disease in a time frame indistinguishable from their rNiV_M_ wild-type strain. In that study, a C^ko^ strain was also completely attenuated with no hamsters showing signs of disease. However, the rNiV_M_ strains examined contained, by design, restriction sites in all intergenic regions, resulting in a rNiV_M_ wild type that was slightly attenuated and not uniformly lethal. This attenuation was critically noted when another group produced wild-type and C^ko^ strains of rNiV_M_ without designed intergenic restriction sites and subsequently infected hamsters^51^. This wild-type rNiV_M_ was uniformly lethal while the C^ko^ strain was partially attenuated with some hamsters surviving. It is likely that the attenuation observed in the first group’s rNiV wild type, and therefore effecting the ko strains, was due to the inserted restriction sites, therefore, partially confounding the hamster *in vivo* data.

While not intending to compare backbones, we were concerned about this potential confounding attenuation; therefore, we produced rNiV_M_-wt, rNiV_M_-W^ko^ and rNiV_M_-V^ko^ strains that do not contain designed restriction sites in the intergenic regions. The multicycle virus growth kinetics in both Vero and 293T (Fig. 2a,b) cell lines were more similar when compared with previous reports^46^,^47^. The rNiV_M_-W^ko^ strain grew to comparable titres as rNiV_M_-wt, with rNiV_M_-V^ko^ growing to slightly lower titres. Pre-treatment of cells with universal IFN-α revealed a marked difference in titre from rNiV_M_-V^ko^-infected cells but no difference between rNiV_M_-wt or rNiV_M_-W^ko^. To the best of our knowledge, these data are the first ever to suggest that the V protein could play a more prominent role than the W protein in regard to the ability of NiV to antagonize the innate immune response in these established cell lines. In addition, these data suggest that the severe attenuation observed in the ferrets challenged with rNiV_M_-V^ko^ was a result of a more robust innate immune response, thereby allowing an effective adaptive immune response to clear the infection when compared with the rNiV_M_-wt and rNiV_M_-W^ko^ cohorts (Fig. 4d).

To further characterize the sensitivity of the recombinants to the innate immune response beyond established cell lines, *in vivo* target cells, primary human endothelial cells of brain or pulmonary origin, were used for multicycle virus growth kinetics (Fig. 2c,d). Interestingly, although rNiV_M_-W^ko^ had similar growth kinetics to rNiV_M_-wt in the brain HBCMECs, it grew to lower titres in pulmonary HPMECs. These *in vitro* data correlate with the *in vivo* data, where we observed severe neurological signs in ferrets infected with rNiV_M_-W^ko^ along with reduced respiratory signs and pathology in the lungs when compared with rNiV_M_-wt. The minimal detectable virus produced from the mutlicycle growth kinetics study in rNiV_M_-V^ko^-infected HBCMECs and HPMECs with intact innate immune responses support the suggestion from the observation made in IFN pre-treated cell lines. In addition, IFN pre-treatment led to stronger inhibition of rNiV_M_-wt replication in HBCMECs and HPMECs when compared with the cell lines examined. This sensitivity to IFN in primary cells, and the fact that NiV W and V proteins have been shown to antagonize both IFN production and IFN-induced signalling, suggests that a reduced inhibition of the innate immune response leads to the altered pathogenesis observed in ferrets infected with rNiV_M_-W^ko^ and rNiV_M_-V^ko^.

Each of the rNiV_M_ strains grew to considerably lower titres in HPMECs and HBCMECs than in the established Vero and 293T cell lines. This is likely because of the fact that established cell lines often have deficiencies in innate immune responses, whereas the primary cells have intact immune responses. By infecting these cells with a low multiplicity of infection (MOI) of 0.01 and observing multicycle growth kinetics, the effects of the innate immune signalling responses can be observed as opposed to cells infected with a high MOI such as observed elsewhere^46^.

All ferrets in the rNiV_M_-wt cohort succumbed within the same time frame observed in previous studies for non-recombinant NiV_M_ (7-8 days p.i.)^33^, demonstrating that the rNiV_M_-wt used in this study is not attenuated compared with non-recombinant NiV_M_. However, ferrets infected with the rNiV_M_-W^ko^ and rNiV_M_-V^ko^ strains exhibited altered disease courses. When compared with the rNiV_M_-wt cohort (Table 1), the ferrets in the rNiV_M_-W^ko^ cohort developed onset of all clinical signs at later time points (∼2–4 days later). Although the rNiV_M_-W^ko^ cohort succumbed to disease, it was at later time points (∼1–3 days). Remarkably, we observed that knocking out the innate immunomodulatory W protein, despite having no effect on disease outcome, dramatically altered the disease course. In the rNiV_M_-W^ko^ cohort respiratory signs and histopathology were less severe, while neurological signs (including seizures) and histopathology were more severe when compared with cohort rNiV_M_-wt. This aligned with the striking observation of NiV antigen detected in neurons (Fig. 7h, black arrow) in the rNiV_M_-W^ko^ cohort. These observations from the rNiV_M_-W^ko^ cohort demonstrate that the W protein does contribute to NiV pathogenesis in ferrets, compared with the study in hamsters^47^. On the basis of our results, the W protein has a novel, yet to be appreciated effect on lung pathogenesis in ferrets, because of its role in antagonizing the inflammatory immune response. While the V protein prominently affects lethal outcome, the W protein appears to contribute to lung pathology with rNiV_M_-W^ko^ resulting in delayed onset of clinical signs, altered disease course and increased time to death. This delay does not appear to be because of any defect inherent in viral replication since rNiV_M_-W^ko^ grows equally well as rNiV_M_-wt in established cell lines as shown in Fig. 2a,b as well as other work^46^,^47^. Rather, this seems to be because of its inability to fully antagonize innate immune cytokine production (as discussed below) thus allowing for decreased replication in HPMECs (Fig. 2d) and the likely influx of innate immune cells due to the cytokine production.

Unlike hamsters that develop either respiratory (high dose) or neurological (low dose) disease dependent on the challenge dose^32^, ferrets appear to either develop both respiratory and neurological disease or no disease at all depending on the challenge dose^26^. However, the numbers involved in that study were small, and we cannot rule out that the apparent control by the innate immune system to rNiV_M_-W^ko^ infection allows for the disease course to be altered, similar to the low dose, neurological disease observed in hamsters. Alternatively, or in combination with this, increased cytokine levels released by rNiV_M_-W^ko^-infected endothelial or other cells in the central nervous system might lead to some of the increase in neurological signs and pathology observed in the rNiV_M_-W^ko^ cohort compared with the rNiV_M_-wt cohort.

The ferrets in the rNiV_M_-V^ko^ cohort developed fever, with some animals also having detectable levels of NiV RNA in whole blood and tissues; however, the lack of respiratory and neurological signs in all animals, except for the transient, mild neurological signs in rNiV_M_-V^ko^-4, was remarkable. This was not due to the lack of virus replication as circulating viral RNA was detected in two of the animals (Fig. 8a), low levels of viral RNA were detected in the tissues of some animals (Fig. 8b) and transient abnormalities occurred in the clinical chemistry and haematological values of some of the animals (Table 1); however, the lack of serious disease in the animals infected with rNiV_M_-V^ko^ demonstrates the prominent role of the V protein in NiV pathogenesis in ferrets. In humans, NiV can cause recrudescence in survivors months to years after the initial infection^19^. It is unknown whether this phenomenon can also occur in ferrets. However, the possibility of this occurring cannot be ruled out for rNiV_M_-V^ko^-infected animals.

While the rNiVs in this study had innate immunomodulatory proteins knocked out, we were interested whether the apparent lack of innate immune control for each led to an effective adaptive immune response in either case. This is of interest because monoclonal antibody therapy has proven effective at preventing NiV-mediated disease in ferrets and non-human primates^26^,^52^. As expected, the animals infected with rNiV_M_-wt did not develop neutralizing antibody before they succumbed (Fig. 4d). Similar results have been described in ferrets infected with NiV_M_ (ref. 33). To date, it is unclear whether the acute time to death prevented the production of neutralizing antibody (7–8 days p.i.) or whether the lack of neutralizing antibodies was due to a NiV-mediated mechanism. The data from the rNiV_M_-W^ko^ cohort revealed that, even with an extended time to death, neutralizing antibodies were not developed after the challenge, even in those surviving to day 10 or 11 p.i. However, all animals infected with rNiV_M_-V^ko^ developed neutralizing antibodies by day 10. Therefore, the innate immunomodulatory V protein of NiV appears to play a role in preventing neutralizing antibody production rather than the short time to death resulting in a lack of neutralizing antibody. Another potential mechanism for the lack of neutralizing antibody production could be because of the stark lymphoid depletion observed in the splenic germinal centres of animals in the rNiV_M_-wt and rNiV_M_-W^ko^ cohorts, but not the rNiV_M_-V^ko^ cohort (Fig. 6c,g,k). This agrees with a similar report of canine distemper virus-infected ferrets where wild-type and C^ko^ viruses were 100% lethal and did not develop neutralizing antibody responses, whereas a V^ko^ virus was attenuated and led to a potent neutralizing antibody response^53^; however, that study did not determine any potential mechanism for this phenomenon.

**Figure 9:**
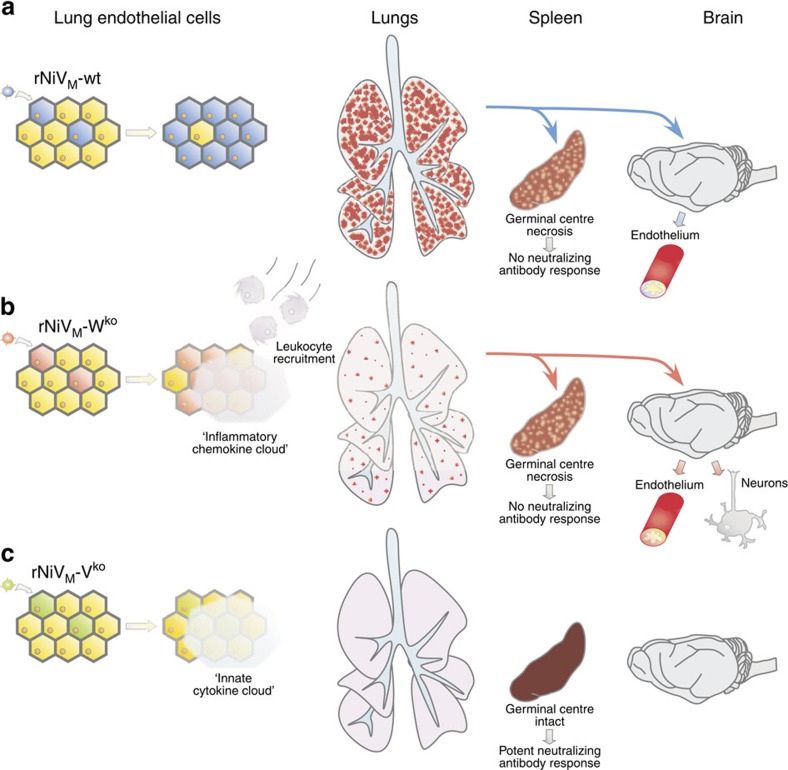
Model of the immunomodulating roles of the NiV W and V proteins in ferret pathogenesis. rNiV_M_-wt (**a**) spreads unhindered in early target lung endothelial cells (Fig. 2d) leading to extensive lung injury (Fig. 5a) followed by spread to the spleen (Fig. 5b) causing germinal centre necrosis (Fig. 6c) leading to a lack of neutralizing antibody response (Fig. 4d); there is also spread to the endothelium of the brain (Fig. 7d) and moderate neurological signs develop before the ferret succumbs to pulmonary disease. rNiV_M_-W^ko^ (**b**) allows lung endothelial cells to generate an ‘inflammatory chemokine cloud’ (Fig. 3 and Supplementary Table 1) somewhat hindering viral titres (Fig. 2d) and presumably allowing increased leukocyte recruitment to the lungs, limiting lung injury (Fig. 5d) and sequestering most viral spread to the endothelium and distinctive inflammatory nodules (Fig. 7f), thus causing milder respiratory signs; rNiV_M_-W^ko^ is still able to spread to the spleen (Fig. 5e) causing germinal centre necrosis (Fig. 7g) and leading to a lack of neutralizing antibody response (Fig. 4d); there is also spread to the brain endothelium and, owing to prolonged time to death, eventually neurons as well leading to severe neurological signs and succumbing to neurological disease. rNiV_M_-V^ko^ (**C**) in unable to grow efficiently in lung endothelial cells (Fig. 2d) possibly due to the production of an ‘innate cytokine cloud’ (Fig. 3 and Supplementary Table 1) and is unable to efficiently spread or cause injury in the lungs (Fig. 5g), spleen (Fig. 5h) or brain (Fig. 7l); the splenic germinal centres remain intact (Fig. 6k) and a potent neutralizing antibody response develops (Fig. 4d) that leads to viral clearance and 100% ferret survival.

While differences in circulating neutralizing antibodies did not appear to be responsible for differences between the rNiV_M_-wt and rNiV_M_-W^ko^ cohorts, we attempted to understand why the rNiV_M_-W^ko^ cohort exhibited less lung pathology than the rNiV_M_-wt cohort by examining the chemokine/cytokine profiles of infected HPMECs, the presumed early target cells in NiV infections. It was notable that many of the pro-inflammatory and leukocyte-attracting cytokines were at higher levels in supernatants from HPMECs infected with rNiV_M_-W^ko^ than in HPMECs infected with either rNiV_M_-wt or rNiV_M_-V^ko^ (Fig. 3 and Supplementary Table 1). Because the HPMECs were infected with a low MOI of 0.01 and presumably rNiV_M_-V^ko^ was unable to spread effectively to other cells (Fig. 2d, green bars), the lower cytokine levels found in many of these supernatants is likely due to only a small proportion of the cells that were being infected and stimulated to produce the cytokines limiting the peak levels. However, both rNiV_M_-wt and rNiV_M_-W^ko^ were presumably able to infect most or all of the cells in the well (Fig. 2d, blue and red bars, respectively) with rNiV_M_-W^ko^ at slightly lower levels. Therefore, the higher levels of chemokines/cytokines produced by the rNiV_M_-W^ko^-infected HPMECs is likely due to the ability of the W protein to suppress and control chemokine/cytokine response in rNiV_M_-wt-infected HPMECs. These data suggest that the rNiV_M_-W^ko^-infected ferret cohort had an increased pulmonary chemokine/cytokine response, allowing for greater leukocyte recruitment to the lungs early in infection diminishing the severe insult to the lungs. In addition, this may explain the increased number and larger inflammatory nodules seen on histopathological examination for the rNiV_M_-W^ko^ cohort (Fig. 7e,f) compared with the rNiV_M_-wt cohort (Fig. 7a,b) and why viral antigen was much more localized to only the endothelium and these inflammatory nodules in the rNiV_M_-W^ko^ cohort. This may have some similarities to what was described with C^ko^ rNiV-infected hamsters^51^, although this leads to some hamsters surviving instead of developing more severe neurological disease as was observed with our rNiV_M_-W^ko^ ferret cohort. We have captured our overall interpretation of these data in Fig. 9.

While we show the V protein is the prominent determinate of pathogenesis (Fig. 9c), strikingly we also show the W protein as a minor determinate of pathogenesis for NiV-mediated disease in ferrets causing a delayed disease course with less severe respiratory signs and lung pathology, as knocking out this protein completely alters the final disease course to the neural tissue (Fig. 9b). This is likely because rNiV_M_-W^ko^ has a reduced ability to suppress the initial chemokine/cytokine response resulting in less severe insult to the lungs allowing for a prolonged disease course for the virus to cause disease within the central nervous system. This is probably the reason for the more severe neurological signs observed in these ferrets. Despite the slower disease course, lymphoid depletion within the splenic germinal centres occurred, no neutralizing antibody response was detected and all animals succumbed to NiV-mediated neurological disease.

Previously, innate immunomodulatory proteins of paramyxoviruses have been studied, including the V and C proteins of measles virus in rhesus macaques^54^, the STAT1-binding domain of the measles virus P and V proteins in rhesus macaques^55^ and the V and C proteins of canine distemper virus in ferrets^53^, without drastically altering the disease course and were touted as potential solutions to vaccines for immunocompromised individuals. We have reported here the first observation of mutating a paramyxovirus immunomodulatory protein, as seen with the W^ko^ NiV recombinant, which alters the disease course but not lethality. On the basis of our findings, further examination of viral innate immunomodulatory proteins is warranted to observe whether this phenomenon is common; this especially should be considered for those who are considering designing vaccines on the basis of attenuating immunomodulatory proteins or therapeutics targeting such proteins found in pathogenic viruses.

Overall, this study reveals the crucial contribution of the NiV V and W proteins to the pathogenesis and disease course in the ferret model with evidence pointing to the V protein being the major determinant with its ability to antagonize the innate immune response in target endothelial cells resulting in attenuated pathogenesis *in vivo*. The W protein on the other hand appears to have the ability to control the chemokine/cytokine response of target endothelial cells, which has an effect on the disease course, although not lethality. The specific mechanisms that the V and W proteins use to contribute to pathogenesis *in vivo* and *in vitro* are yet to be elucidated along with the contribution of other possible pathogenic determinates. These include the role of the C protein in pathogenesis in comparison with the data available from the hamster model, and the contribution of the STAT1-binding domain shared by the P, V and W proteins. Studies looking deeper into these mechanisms are warranted and necessary to ultimately understand how NiV evades the host immune response contributing to pathogenesis.

## Methods

### Cell lines

BSR-T7/5 cells, a BHK-21 cell line stably expressing T7 RNA polymerase^56^, were maintained in Dulbecco’s modified Eagle medium (DMEM; Gibco, Carlsbad, CA) supplemented with 10% fetal bovine serum (FBS; Gibco), 100 U ml^−1^ penicillin, 100 g ml^−1^ streptomycin and 0.5 mg ml^−1^ Geneticin (Gibco). Vero 76 cells (ATCC CRL-1587) were maintained in Eagle’s Minimum Essential Medium supplemented with 10% FBS and 100 U ml^−1^ penicillin (Gibco), 100 g ml^−1^ streptomycin (Gibco). HEK 293T/17 cells (ATCC CRL-11268) were maintained in DMEM supplemented with 10% FBS, 100 U ml^−1^ penicillin and 100 g ml^−1^ streptomycin.

### Primary cells

HPMECs (Lonza, Basel, Switzerland) were maintained in EBM-2MV media supplemented with 5% FBS and proprietary concentrations of human epidermal growth factor, fibroblast growth factor, vascular endothelial growth factor, insulin-like growth factor, hydrocortisone and gentamicin, all provided by Lonza. HBCMECs were obtained from Sciencell (Carlsbad, CA) and were maintained in EBM-2 containing 5% FBS, 1.4 μM hydrocortisone, 5 μg ml^−1^ ascorbic acid, 1 ng ml^−1^ basic fibroblast growth factor (Sigma, St Louis, MO), 1X chemically defined lipid concentrate, 10 mM HEPES and Penicillin–Streptomycin (Life Technologies, Carlsbad, CA). Media were changed every 2–3 days and cells were used up to passage 7.

### Plasmid construction and generation of recombinant NiVs

The NiV genomic sequence used to construct the rNiVs in this study was UMMC1 (GenBank accession no. AY029767), an isolate cultured from the cerebrospinal fluid of an encephalitic human patient in the initial outbreak in Malaysia. This NiV_M_ genome was assembled into three segments, A (nt 1–6,780), B (nt 6,780–10,404) and C (nt 10,404–18,246), as described previously^43^. These fragments could then be mutated followed by assembly into full-length cDNA clones in pSL1180 cloning vectors containing T7 promoter and terminator sequences and a hepatitis delta virus ribozyme sequence. Three NiV_M_ full-length cDNA clones (pFL-NiV_M_-wt, pFL-NiV_M_-W^ko^ and pFL-NiV_M_-V^ko^) were constructed. Site-directed mutagenesis was performed by Mutagenex Inc (Piscataway, NJ) to introduce the W^ko^ (c3628t) and V^ko^ (a3629t) mutations in the A segment (Fig. 1b). These mutations introduced stop codons shortly after the editing site in the W and V open reading frames, respectively.

The helper plasmid pTM1-HA NiV_M_ P was constructed as described previously^43^; the P gene was haemagglutinin (HA)-tagged at the amino terminus and subcloned into the pTM1 expression plasmid. The helper plasmids pTM1.W-NiV_M_ N and pTM1.W-NiV_M_ L were constructed by amplifying the sequences for the N and L genes from the A and C segments, respectively, with PCR (Supplementary Table 2) using Platinum PFX polymerase (Life Technologies) and were cloned into a pTM1.W expression vector.

BSR-T7/5 cells were seeded in six-well plates and were co-transfected with 3.5 μg of NiV_M_ full-length cDNA clone, 0.2 μg of pTM1-HA NiV_M_ P, 0.75 μg of pTM1.W-NiV_M_ N and 0.4 μg of pTM1.W-NiV_M_ L per well in Optimem (Gibco) with Lipofectamine 2,000 (Life Technologies) according to the manufacturer’s protocol. At 72 h post transfection, the medium and cells were collected and passaged on Vero cells. Cytopathic effect (CPE) was typically observed to be beginning between days 4 and 8 p.i. The medium was then collected and passed on Vero cells for plaque purification of the virus. A small quantity (P1) of the plaque-purified virus was then grown in Vero cells followed by a larger quantity (P2) in Vero cells infected with a MOI of 0.01. At 48 h p.i. the virus-containing medium was harvested, clarified by low-speed centrifugation, aliquoted and stored at −80 °C. The presence of introduced mutations was confirmed by sequencing of RT–PCR fragments amplified from virus RNA isolated from virus stocks with Trizol LS (Ambion, Carlsbad, CA). Virus titres were determined by standard plaque assay using 5% neutral red. All experiments using full-length clones or infectious rNiV_M_ were performed using protocols approved by the Institutional Biosafety Committee (IBC) and National Institutes of Health recombinant advisory committee (NIH RAC) in BSL-4 containment at the Galveston National Laboratory in Galveston, TX.

### Western blot analysis

Polyclonal rabbit antisera against the unique C-terminal domains of the NiV P, W and V proteins and to the NiV C protein were produced by GenScript (Piscataway, NJ). Overall, 1.2 × 10^6^ Vero cells per well were seeded in a six-well plate and infected with rNiV_M_-wt, rNiV_M_-W^ko^ or rNiV_M_-V^ko^ at an MOI of 0.01. The cells were harvested at 40 h.p.i. in 1 ml of 2 × Laemmli sample buffer (Bio-Rad, Hercules, CA) and heated to 95 °C for 20 min. Samples were then run on a denaturing 4–12% SDS–PAGE gel (Bio-Rad). Proteins were transferred from the gel on polyvinilidene fluoride (PVDF) membranes and blocked in TTBS (100 mM Tris-HCl pH 7.5, 0.9% NaCl, 0.1% Tween 20) with 5% skim milk. PVDF membranes were incubated with polyclonal rabbit antisera against P, V, W and C described above, diluted in TTBS with 5% milk (P: 1:10,000; V: 1:5,000; W: 1:5,000; C: 1:500) for 1 h at room temperature and washed three times in TTBS. The membranes were then incubated with anti-rabbit IgG conjugated to horseradish peroxidase (Sigma-Aldrich; 1:20,000 dilution) for 1 h at room temperature, washed three times in TTBS, incubated with ECL reagent (Promega) for 5 min and imaged with a VersaDoc (Bio-Rad).

### Virus growth kinetics

Overall, 1.2 × 10^6^ cells per well of 293T or Vero cells were seeded in six-well plates and incubated at 37 °C for 12 h with either regular medium, or medium containing 1,000 U ml^−1^ of Universal IFN-α (PBL Assay Science, Piscataway, NJ). The cells were then infected at an MOI of 0.01 with rNiV_M_-wt, rNiV_M_-W^ko^ or rNiV_M_-V^ko^ for 1 h followed by the removal of the inoculum, four washes with PBS and the addition of fresh medium. Supernatants were collected at 1, 6, 12, 24, 36, 48 and 72 h p.i., clarified by centrifugation, aliquoted and stored at −80 °C. All infections were performed in duplicate. Samples were then titred on Vero cells using standard plaque assays. Limit of detection was 25 p.f.u. per ml.

Similarly, 2.5 × 10^5^ cells per well of HPMEC or HBCMEC were seeded in 24-well plates and were allowed to polarize for 48 h at 37 °C, followed by incubation at 37 °C for 12 h with either regular medium, or medium containing 1,000 U ml^−1^ of Universal IFN-α (PBL). The cells were then infected at an MOI of 0.01 with rNiV_M_-wt, rNiV_M_-W^ko^ or rNiV_M_-V^ko^ for 1 h followed by the removal of the inoculum, four washes and the addition of fresh medium. Supernatants were collected at 1, 24, 48 and 72 h p.i., clarified by centrifugation, aliquoted and stored at−80 °C. Additional aliquots were stored for use in chemokine/cytokine analysis described below. All infections were performed in duplicate. Samples were then titred on Vero cells using standard plaque assays. Limit of detection was 25 p.f.u. per ml.

### Chemokine/cytokine analysis

HPMECs were infected and supernatants aliquoted and stored as described above. Levels of 6Ckine/CCL21, BCA-1/CXCL13, CTACK/CCL27, ENA-78/CXCL5, Eotaxin/CCL11, Eotaxin-2/CCL24, Eotaxin-3/CCL26, Fractalkine/CX3CL1, GCP-2/CXCL6, GM-CSF, Gro-α/CXCL1, Gro-β/CXCL2, I-309/CCL1, IFN-γ, IL-1β, IL-2, IL-4, IL-6, IL-8/CXCL8, IL-10, IL-16, IP-10/CXCL10, I-TAC/CXCL11, MCP-1/CCL2, MCP-2/CCL8, MCP-3/CCL7, MCP-4/CCL13, MDC/CCL22, MIF, MIG/CXCL9, MIP-1α/CCL3, MIP-1δ/CCL15, MIP-3α/CCL20, MIP-3β/CCL19, MPIF-1/CCL23, SCYB16/CXCL16, SDF-1α+β/CXCL12, TARC/CCL17, TECK/CCL25 and TNF-α were quantified in supernatants from infected HPMECs. Briefly, gamma-irradiated supernatants were diluted to 1:4, and 50 μl of each sample was quantified using a Bio-Plex Pro Human Chemokine Panel, 40-Plex (Bio-Rad) according to the manufacturer’s instructions. Infections and supernatant sample collection were performed in duplicate, and each sample was quantified in duplicate. Samples were assayed across at least a 100-bead region performed on the Bio-Plex-200 machine and analysed using the Bio-Plex Manager Software (v 6.1; Bio-Rad).

Levels of IFN-α and IFN-β were quantified using 25 μl of undiluted supernatant in either a Human IFN alpha (Multi-Subtype) ELISA kit or Human IFN beta ELISA kit, respectively (Thermo Scientific, Waltham, MA) according to the manufacturer’s instructions. All samples were read for dilution end points at 405 nm on a Molecular Devices Emax system microplate reader (Molecular Devices, Sunnyvale, CA).

### Statistics

Conducting animal studies in BSL-4 severely restricts the number of animal subjects, the volume of biological samples that can be obtained and the ability to repeat assays independently and thus limit statistical analysis. Consequently, data are presented as the mean calculated from replicate samples, not replicate assays, and error bars represent the s.d. across replicates.

The Prism 5 software was used to calculate statistical significance throughout this study using the following tests: log-rank (Mantel–Cox) test for Kaplan–Meier survival curves; analysis of variance (ANOVA) with Dunnett’s multiple comparison test for viral growth kinetics and chemokine/cytokine analysis.

### Animals

Animal studies were performed in BSL-4 biocontainment at the GNL at UTMB in Galveston and were approved by the UTMB Institutional Animal Care and Use Committee (IACUC). Animal research was conducted in compliance with the Animal Welfare Act and other Federal statutes and regulations relating to animals and experiments involving animals, and adheres to the principles stated in the eighth edition of the *Guide for the Care and Use of Laboratory Animals*, National Research Council, 2011 (ref. 57). The facility where this research was conducted is fully accredited by the Association for Assessment and Accreditation of Laboratory Animal Care International.

Fifteen female, 12-month-old ferrets (*Mustela putorius furo*) weighing 0.75–1.0 kg were housed in groups of two (pilot animals) or three animals per virus cohort. Before infection, subjects were anaesthetized by 5% isofluorane and had transponder chips (BioMedic Data Systems, Seaford, DE) implanted subcutaneously for animal identification and temperature monitoring. For challenge and procedures, animals were anaesthetized with a ketamine acepromazine xylazine cocktail and inoculated i.n. with ∼5,000 p.f.u. of rNiV_M_-wt, rNiV_M_-W^ko^ or rNiV_M_-V^ko^ in 0.5 ml of 10% FBS Hank’s Balanced Salt Solution (Gibco). Animals were anaesthetized for clinical examination, respiration quality and blood collection on days 0, 6 and 35 p.i. or terminal end point collection for pilot animals, and days 0, 3, 6, 15 and 35 p.i. or terminal end point for all other animals (Fig. 4a). After the challenge, animals were assessed daily for weight and temperature, and scored on a scale of 0–12 for clinical observations on the basis of coat appearance, social behaviour and provoked behaviour; animals scoring 9 or more were euthanized as per the IACUC protocol. Subjects in the rNiV_M_-V^ko^ cohort were euthanized at the study end point on day 35 p.i., whereas the subjects in the rNiV_M_-wt and rNiV_M_-W^ko^ cohorts reached euthanasia criteria and were euthanized according to approved humane end points on days 7–11 p.i.

### rNiV_M_ serum neutralization assays

PRNT_50_s were determined using a conventional serum neutralization assay. Briefly, sera were serially diluted twofold and incubated with ∼100 p.f.u. of rNiV_M_-wt for 1 h at 37 °C. Virus and antibodies were then added to individual wells of six-well plates of confluent Vero cell monolayers in duplicate. Plates were stained with neutral red 2 days after infection and plaques were counted 24 h after staining. The 50% neutralization titre (PRNT_50_) was determined as the serum dilution at which there was a 50% reduction in plaque counts versus control wells.

### Specimen collection and processing in rNiV_M_-infected ferrets

Blood was collected and placed in MiniCollect EDTA tubes or serum tubes (Greiner Bio One, Monroe, NC). Immediately following sampling, 100 μl of whole blood was added to 600 μl of AVL viral lysis buffer with carrier RNA (Qiagen) for RNA extraction. For tissues, ∼100 mg was stored in 1 ml RNAlater (Qiagen) for 96 h to stabilize RNA. RNAlater was completely removed, and tissues were homogenized in 600 μl RLT buffer (Qiagen) in a 2-ml cryovial using a tissue lyser (Qiagen) and 1.4-mm ceramic beads (Precellys, Saint-Quentin-en-Yvelines, France). The tissue samples included the right lung upper lobe, right lung middle lobe, right lung lower lobe, left lung upper lobe, left lung middle lobe, left lung lower lobe, liver, spleen, kidney, adrenal gland, pancreas and brain (frontal cortex). All whole-blood samples were inactivated in AVL viral lysis buffer with carrier RNA, and tissue samples were homogenized and inactivated in RLT buffer before removal from the BSL-4 laboratory. Subsequently, RNA was isolated from whole blood and swabs using the QIAamp viral RNA kit (Qiagen) from tissues using the RNeasy minikit (Qiagen) according to the manufacturer’s instructions supplied with each kit.

### Haematology and serum biochemistry

Blood was collected on days 0, 6 and 35 p.i. or on terminal end point for pilot animals, and days 0, 3, 6, 15 and 35 p.i. or on terminal end point for all other animals (Fig. 4a). Complete blood counts of total white blood cell counts, white blood cell differentials, red blood cell counts, platelet counts, haematocrit values, total haemoglobin concentrations, mean cell volumes, mean corpuscular volumes and mean corpuscular haemoglobin concentrations were analysed from blood collected in MiniCollect EDTA tubes (Greiner Bio One) using a Hemavet HV950FS instrument as per the manufacturer’s instructions (Drew Scientific, Oxford, CT). Serum was centrifuged at 400*g* (rcf) for 10 min, and analysis of blood chemistries was performed using a VetScan classic analyser and comprehensive diagnostic profile rotors measuring of albumin, amylase, alanine aminotransferase, alkaline phosphatase, calcium, glucose, total protein, total bilirubin, blood urea nitrogen, creatinine, phosphorus, sodium and total protein (Abaxis, Union City, CA). All blood and serum samples were processed and analysed immediately after collection.

### Histopathology and IHC

Necropsy was performed on all subjects. Tissue samples of all major organs were collected for histopathologic and immunohistochemical examination and were immersion-fixed in 10% neutral buffered formalin for at least 21 days in BSL-4. Subsequently, formalin was changed; specimens were removed from BSL-4, processed in BSL-2 by conventional methods and embedded in paraffin and sectioned at 5-μm thickness as previously described^33^. Briefly, for IHC, specific anti-NiV immunoreactivity was detected using an anti-NiV N protein rabbit polyclonal primary antibody^28^ (kindly provided by Dr Christopher Broder, Uniformed Services University of the Health Sciences, Bethesda, MD) at a 1:5,000 dilution for 30 min. The tissue sections were processed for IHC using the Dako Autostainer (Dako, Carpinteria, CA). Secondary antibody used was biotinylated goat anti-rabbit IgG (Vector Laboratories, Burlingame, CA) at 1:200 for 30 min followed by Dako LSAB2 streptavidin-HRP (Dako) for 15 min. Slides were developed with Dako DAB chromagen (Dako) for 5 min and counterstained with haematoxylin for 1 min. Non-immune rabbit IgG was used as a negative staining control.

### Detection of rNiV_M_ load

RNA was isolated from whole blood or tissues and analysed using primers/probe targeting the N gene and intergenic region between N and P of NiV for qRT–PCR with the probe used here being 6-carboxyfluorescein-5′-CGTCACACATCAGCTCTGACGA-3′-6 carboxytetramethylrhodamine (Life Technologies). rNiV RNA was detected using the CFX96 detection system (Bio-Rad) in One-step probe qRT-PCR kits (Qiagen) with the following cycle conditions: 50 °C for 10 min, 95 °C for 10 s and 40 cycles of 95 °C for 10 s and 59 °C for 30 s. Threshold cycle (*C*_t_) values representing rNiV genomes were analysed with the CFX Manager Software, and data are shown as GEq. To create the GEq standard, RNA from NiV challenge stocks was extracted and the number of NiV genomes was calculated using Avogadro’s number and the molecular weight of the NiV genome. Virus titration was performed by plaque assay with Vero cells from all whole blood and tissue samples. Briefly, increasing 10-fold dilutions of the samples were adsorbed to Vero cell monolayers in duplicate wells (200 μl); the limit of detection was 25 p.f.u. per ml.

## 

## Additional information

**How to cite this article:** Satterfield, B. *et al.* The immunomodulating V and W proteins of Nipah virus determine disease course. *Nat. Commun.* 6:7483 doi: 10.1038/ncomms8483 (2015).

## Supplementary Material

Supplementary Information Supplementary Figure 1 and Supplementary Tables 1-2

## Figures and Tables

**Table 1:** Clinical disease in ferrets after experimental infection with rNiV_M_.

**Ferret no.**	**Clinical outcome**	**Resp** a	**Neuro** b	**Hem** c	**Fever**	**Clinical disease**
rNiV_M_-wt-1	F.D. d8	**+**	**++**	**−**	d4–7	Thrombocytopaenia (d6); hypoalbuminemia (d6); hyperglycaemia (d6); depression (d6–7); lethargy (d6–7); inappetence (d7); ocular and nasal discharge (d7); myoclonus (d7); ataxia (d7); loss of 20% body weight
rNiV_M_-wt-2	EU d8	**+**	**−**	**−**	d4–7	Thrombocytopaenia (d6,8); lymphopaenia (d6,8); hypoalbuminemia (d6,8); >3-fold increase in BUN (d8); hyperglycaemia (d6); depression (d6–8); lethargy (d6–8); sneezing (d7–8); ocular, nasal and oral discharge (d7–8); severely obtunded (d8)
rNiV_M_-wt-3	F.D. d8	**+**	**++**	**−**	d5–6	Thrombocytopaenia (d6); lymphopaenia (d6); hypoalbuminemia (d6); hyperglycaemia (d6); depression (d5–7); lethargy (d5–7); inappetence (d6–7); (d7); ocular and nasal discharge (d6–7); ataxia (d7); hind–limb myoclonus (d7)
rNiV_M_-wt-4	EU d7	**+**	**++**	**+**	d4–6	Thrombocytopaenia (d6–7); lymphopaenia (d6–7); hypoalbuminemia (d6,7); >3-fold increase in BUN (d7); hyperglycaemia (d7); depression (d6–7); lethargy (d5–7); inappetence (d6–7); ocular and nasal discharge (d6–7); nasal/oral frothing (d7); periorbital/facial oedema (d7); ataxia (d6–7); myoclonus (d7); violent tremors (d7); hypothermia (d7)
rNiV_M_-wt-5	EU d8	**+**	**+**	**+**	d4–7	Thrombocytopaenia (d6,8); lymphopaenia (d6); hypoalbuminemia (d6,8); >3-fold increase in BUN (d8); depression (d5–7); lethargy (d5–7); inappetence (d6–8); dehydration (d8); rales (d6–8); ocular, nasal and oral discharge (d6); ataxia (d6–8); severe hypothermia (d8)
rNiV_M_-W^ko^-1	EU d9	**+**	**+++**	**−**	d7–8	Thrombocytopaenia (d9); lymphopaenia (d9); hypoalbuminemia (d9); >3-fold increase in BUN (d6,9); hyperglycaemia (d9); depression (d8–9); lethargy (d7–9); dehydration (d9); sneezing (d8–9); rales (d9); nasal and oral frothing (d9); severe ataxia (d9); periodic seizures (d9); hypothermia (dy9)
rNiV_M_-W^ko^-2	EU d10	**+**	**+++**	**−**	d6–9	Thrombocytopaenia (d10); hypoalbuminemia (d10); >3-fold increase in BUN (d10); depression (d8–10); lethargy (d7–10); inappetence (d8–10); hypersalivation (d10); sneezing (d8); ataxia (d8–10); full-body tremors (d10); periodic severe seizures with random vocalization (d10); severe hypothermia (d10)
rNiV_M_-W^ko^-3	EU d11	**+**	**−**	**+**	d7–9	Thrombocytopaenia (d10–11); lymphopaenia (d10); hypoalbuminemia (d10,11); >3-fold increase in BUN (d11); hyperglycaemia (d10); depression (d8–11); lethargy (d7–11); sneezing (d8–10); ocular and nasal discharge (d10–11); severe hypothermia (d11); obtunded (d11)
rNiV_M_-W^ko^-4	EU d8	**+**	**++**	**−**	d6–8	Thrombocytopaenia (d8); lymphopaenia (d8); hypoalbuminemia (d8); depression (d7–8); lethargy (d7–8); sneezing (d7–8); nasal discharge (d7); heavy nasal and oral frothing (d8); severe ataxia (d8); severely obtunded (d8)
rNiV_M_-W^ko^-5	EU d9	**+**	**+++**	**+**	d6–8	Thrombocytopaenia (d6, 9); lymphopaenia (d9); hypoalbuminemia (d9); >3-fold increase in BUN (d9); hyperglycaemia (d9); depression (d8–9); lethargy (d7–9); dehydration (d9); sneezing (d7–8); rales (d9); nasal discharge (d7); nasal and oral frothing (d9); severe ataxia (d9)
rNiV_M_-V^ko^-1	s.e. d35	**−**	**−**	**−**	d6–9	lymphopaenia (d6)
rNiV_M_-V^ko^-2	s.e. d35	**−**	**−**	**−**	d6–10	Thrombocytopaenia (d6); lymphopaenia (d6); lethargy (d8, 11)
rNiV_M_-V^ko^-3	s.e. d35	**−**	**−**	**−**	d6–7	Thrombocytopaenia (d6); lymphopaenia (d6)
rNiV_M_-V^ko^-4	s.e. d35	**−**	**−**	**−**	d7–11	lymphopaenia (d6,10,15); hypoalbuminemia (d6,10,15); hyperglycaemia (d15); depression (d12–16); lethargy (d12–16); nasal discharge (d12); tremors in head and neck with occasional facial and ear twitching (d12–23); mild ataxia (d19–23)
rNiV_M_-V^ko^-5	s.e. d35	**−**	**−**	**−**	d6–10	Thrombocytopaenia (d6,10); lymphopaenia (d10); hypoalbuminemia (d10)

BUN, blood urea nitrogen; EU, euthanized due to rNiV_M_-mediated disease; d, day p.i.; F.D., found dead; hem, hemorrhage; neuro, neurologic involvement; resp, respiratory involvement; s.e., euthanized at study end point.

^a^The absence (**−**) or presence (**+**) of increased respiratory effort and/or rate.

^b^The absence (**−**) or presence of minor (**+**), moderate (**++**) or severe (**+++**) neurological signs.

^c^Extensive perioribtal, facial and ventral neck oedema with subcutaneous haemorrhages.
