# Supplementary material for: The immunomodulating V and W proteins of Nipah virus determine disease course
Source: Nat Commun. 2015 Jun 24;6:7483. doi: 10.1038/ncomms8483 (PMC4482017; doi:10.1038/ncomms8483)
Supplement: Supplementary Information — Supplementary Figure 1 and Supplementary Tables 1-2 [file ncomms8483-s1.pdf]

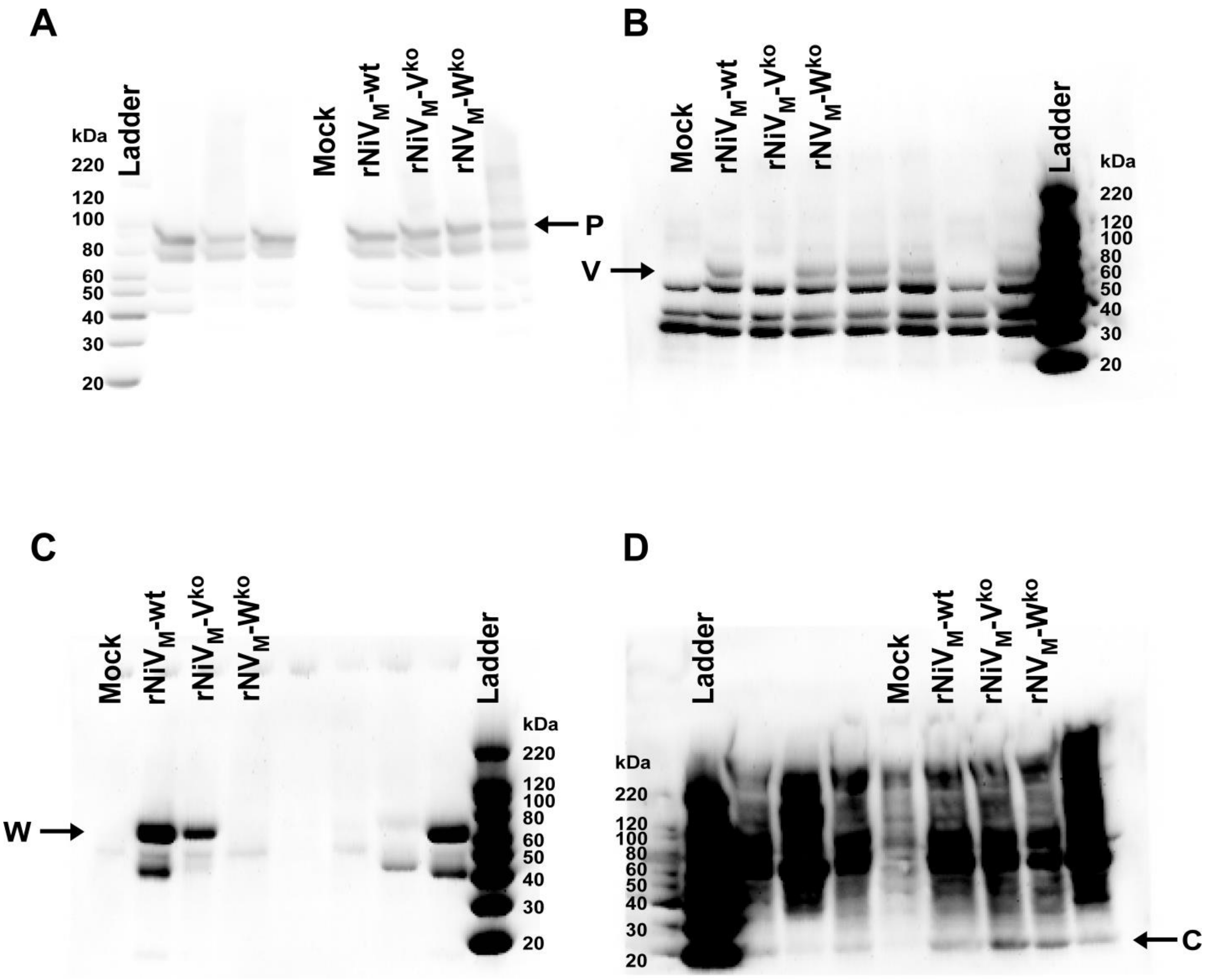

Supplementary Figure 1. Complete Western blots of rNiVs. Western blot analysis of Vero cell lysates either mock-infected, or infected with rNiV$_{M}$-wt, rNiV$_{M}$-V^{ko}, or rNiV$_{M}$-W^{ko} in the lanes indicated. NiV P, V, W, and C specific polyclonal antibodies were used to detect the presence or absence of the respective proteins. Non-specific binding is also observed. The MagicMark™ XP Western Protein Standard is used for the ladder and sizes are shown in kDas. Arrows indicated the size of the P (~85 kDa), V (~55 kDa), W (~54 kDa), and C (~20 kDa) proteins as indicated.

Supplementary Table 1. Chemokine/Cytokine levels in supernatants of infected primary human pulmonary microvascular endothelial cells (HPMEC)

		$rNiV_M$-wt				$rNiV_M$-W^{ko}				$rNiV_M$-V^{ko}			
**Chemokine /Cytokine**	**CCL/CXCL designation**	**1 hpi**	**24 hpi**	**48 hpi**	**72 hpi**	**1 hpi**	**24 hpi**	**48 hpi**	**72 hpi**	**1 hpi**	**24 hpi**	**48 hpi**	**72 hpi**
**Inflammatory chemokines**													
ENA-78	CXCL5	494.0 ±9.6	741.3 ±166.4	776.1 ±176.9	771.8 ±364.0	**259.8 ±10.0**	960.8 ±301.7	**1367.3 ±85.1**	**1382.2 ±374.7**	453.1 ±9.6	630.7 ±106.6	880.9 ±169.1	1116.1 ±102.7
Eotaxin	CCL11	6.9 ±1.2	12.3 ±1.7	20.0 ±1.1	23.2 ±0	**1.7 ±1.3**	11.1 ±3.5	**44.3 ±1.1**	**34.9 ±3.4**	**3.4 ±1.2**	9.4 ±1.3	20.8 ±4.5	**33.1 ±3.9**
Fractalkine	CX3CL1	9.6 ±1.3	20.7 ±6.3	127.0 ±0.9	143.8 ±7.1	**2.6 ±0.3**	**29.8 ±0.7**	**408.3 ±13.8**	**547.9 ±170.7**	**3.0 ±0.6**	**11.1 ±7.2**	138.2 ±11.4	**315.1 ±57.4**
Gro-α	CXCL1	36.4 ±9.9	840.4 ±357.0	1704.4 ±397.4	2326.7 ±51.0	**16.2 ±2.6**	**1447.5 ±122.2**	**3794.7 ±319.0**	3529.2 ±1493.2	36.7 ±4.9	456.4 ±279.8	1418.6 ±347.5	2317.9 ±389.8
Gro-β	CXCL2	---	27.8 ±29.3	79.7 ±18.3	122.4 ±13.3	---	**73.2 ±15.6**	**200.0 ±12.8**	**202.7 ±62.2**	---	24.4 ±9.1	86.5 ±25.9	146.8 ±27.5
IL-8*	CXCL8	10.7 ±2.6	2800.2 ±194.7	3808.9 ±268.1	4054.0 ±451.1	**35.5 ±7.8**	**4352.3 ±495.0**	**5794.4 ±121.5**	**7830.8 ±1445.7**	29.9 ±17.8	2481.7 ±544.7	4268.9 ±270.0	5333.7 ±158.9
IP-10	CXCL10	8.9 ±1.3	23.9 ±1.4	2200.3 ±116.3	2325.9 ±174.1	**5.0 ±4.2**	**39.6 ±12.7**	**6957.7 ±1072.6**	**7266.3 ±1190.9**	13.9 ±1.9	15.6 ±8.1	3160.1 ±479.7	**10517.6 ±265.8**
I-TAC*	CXCL11	9.0 ±6.4	30.1 ±10.3	816.3 ±60.5	1106.3 ±8.8	4.0 ±0.7	**61.3 ±14.5**	**3086.8 ±64.0**	**4321.5 ±791.3**	1.0 ±0.1	16.8 ±6.7	978.2 ±10.8	**2142.3 ±271.2**
MCP-1*	CCL2	11.9 ±4.4	1246.7 ±289.4	2452.0 ±85.6	2870.2 ±321.5	11.3 ±6.7	**1885.6 ±78.8**	**4527.9 ±227.5**	**7309.3 ±1524.5**	8.8 ±1.2	839.9 ±378.9	**2978.2 ±517.8**	**4495.3 ±272.6**
MCP-4	CCL13	2.3 ±0.1	22.4 ±2.6	52.2 ±1.0	60.1 ±2.6	1.6 ±0.1	**33.7 ±1.2**	**94.0 ±6.5**	89.5 ±39.3	1.9 ±0.5	**15.9 ±5.2**	48.7 ±5.2	82.5 ±25.6
MIP-1δ	CCL15	11.9 ±3.5	35.6 ±9.8	462.0 ±15.6	802.3 ±20.8	**5.0 ±0.8**	**51.9 ±4.6**	**806.6 ±229.4**	**1322.1 ±154.2**	8.1 ±1.1	26.5 ±7.8	577.5 ±16.8	**3285.7 ±1632.9**
MIP-3α	CCL20	3.6 ±0.5	5.4 ±2.6	14.6 ±1.0	13.5 ±1.0	0.4 ±0.2	6.2 ±0.5	**26.3 ±4.8**	18.9 ±11.6	2.7 ±1.8	4.3 ±2.3	10.4 ±2.5	11.8 ±4.5
MPIF-1	CCL23	---	26.2 ±2.8	53.9 ±4.2	61.0 ±6.0	---	22.9 ±3.8	**106.8 ±3.5**	**110.3 ±8.7**	---	16.6 ±5.2	70.1 ±12.4	**88.4 ±6.5**
SCYB16	CXCL16	1.9 ±1.0	8.3 ±0.3	25.4 ±6.2	37.5 ±2.8	---	7.7 ±0.3	**62.8 ±2.3**	**137.3 ±16.3**	1.0 ±0.1	4.7 ±2.9	21.8 ±4.8	**63.0 ±6.7**
SDF-1α+β	CXCL12	27.1 ±26.7	29.8 ±12.5	17.1 ±12.5	8.2 ±1.2	---	14.6 ±9.0	57.5 ±6.7	69.0 ±15.6	8.2 ±1.4	---	27.1 ±26.7	23.4 ±3.5
6Ckine	CCL21	---	74.4 ±1.4	336.2 ±22.7	311.9 ±11.7	---	---	510.9 ±57.2	417.6 ±109.6	---	---	285.7 ±48.7	422.6 ±144.8
Eotaxin-2	CCL24	95.8 ±10.4	64.1 ±21.5	24.7 ±1.8	36.1 ±2.2	28.0 ±11.4	14.5 ±1.6	20.6 ±23.6	53.1 ±18.5	56.4 ±3.5	20.7 ±9.3	17.8 ±1.3	---
Eotaxin-3	CCL26	5.1 ±0.8	4.7 ±3.1	6.0 ±2.0	9.3 ±1.1	---	---	5.7 ±0.3	33.6 ±8.0	---	---	0.7 ±0.1	0.7 ±0.2
GCP-2	CXCL6	---	6.2 ±0.7	10.9 ±5.2	10.1 ±2.1	---	8.8 ±1.1	18.8 ±1.9	34.1 ±17.9	---	3.8 ±0.3	12.2 ±3.4	16.0 ±0.7
MIG	CXCL9	---	12.4 ±1.2	13.9 ±5.9	13.3 ±5.2	---	---	11.7 ±0.1	22.1 ±12.9	---	---	9.4 ±4.3	17.0 ±1.5
MIP-3β	CCL19	20.6 ±6.0	27.9 ±1.4	59.7 ±24.7	49.4 ±10.1	3.3 ±0.8	31.8 ±6.9	77.9 ±3.6	64.8 ±5.3	14.1 ±2.1	21.8 ±4.5	43.1 ±3.9	76.3 ±6.0
TECK	CCL25	32.8 ±16.4	128.9 ±7.9	585.3 ±190.1	580.0 ±136.8	---	87.0 ±19.9	510.1 ±22.8	550.8 ±80.7	29.9 ±20.6	148.4 ±3.9	488.6 ±38.1	623.0 ±68.7
BCA-1	CXCL13	---	---	---	---	---	---	---	---	---	---	---	---
CTACK	CCL27	---	---	---	---	---	---	---	---	---	---	---	---
I-309	CCL1	---	---	---	---	---	---	---	---	---	---	---	---
MCP-2	CCL8	---	---	---	---	---	---	---	---	---	---	---	---
MCP-3	CCL7	---	---	---	---	---	---	---	---	---	---	---	---

MDC	CCL22	---	---	---	---	---	---	---	---	---	---	---	---
MIP-1α	CCL3	---	---	---	---	---	---	---	---	---	---	---	---
TARC	CCL17	---	---	---	---	---	---	---	---	---	---	---	---
**Innate immune cytokines**													
TNF-α	N/A	2.1 ±2.2	2.5 ±1.1	10.7 ±1.6	11.9 ±1.6	---	1.7 ±0.2	**37.8 ±1.3**	**36.9 ±13.0**	2.0 ±0.4	1.1 ±0.8	11.7 ±2.4	**22.8 ±3.0**
IL-6	N/A	0.2 ±0.2	11.0 ±2.9	41.0 ±8.9	43.9 ±2.3	---	**23.7 ±1.5**	**119.1 ±9.6**	**116.2 ±41.3**	0.3 ±0.1	7.5 ±4.5	36.2 ±9.8	**59.3 ±4.1**
IL-10	N/A	2.6 ±0.3	13.1 ±0.7	25.2 ±1.1	31.2 ±4.5	---	**17.9 ±1.3**	**40.1 ±3.7**	34.3 ±10.0	---	**9.4 ±0.5**	24.2 ±0.2	34.6 ±2.4
GM-CSF	N/A	66.5 ±13.9	55.8 ±2.1	44.7 ±19.5	39.4 ±21.9	56.4 ±9.6	50.8 ±7.5	53.7 ±3.4	72.9 ±12.7	52.0 ±5.0	44.9 ±4.2	44.6 ±5.4	45.8 ±4.6
MIF*	N/A	223.2 ±57.5	496.5 ±114.6	2419.3 ±632.9	2300.2 ±444.6	188.2 ±17.2	427.8 ±0.4	1776.7 ±189.3	2285.8 ±475.5	292.8 ±29.7	680.2 ±33.7	2069.8 ±177.1	2298.0 ±215.7
IFN-α	N/A	---	---	---	---	---	---	---	---	---	---	---	---
IFN-β	N/A	---	---	---	---	---	---	---	---	---	---	---	---
IFN-γ	N/A	---	---	---	---	---	---	---	---	---	---	---	---
IL-1β	N/A	---	---	---	---	---	---	---	---	---	---	---	---
IL-2	N/A	---	---	---	---	---	---	---	---	---	---	---	---
IL-4	N/A	---	---	---	---	---	---	---	---	---	---	---	---
IL-16	N/A	---	---	---	---	---	---	---	---	---	---	---	---

Reported as mean ± standard deviation

All values reported in pg/ml except: * indicates IL-8, I-TAC, MCP-1, and MIF are reported as fluorescence intensity because values exceeded the upper limits of the standard curve.

Values in **BOLD** have a p-value < 0.05 when compared to $rNiV_M$-wt

N/A, not applicable

--- indicates that the value was below the detection threshold

Supplementary Table 2. Cloning primers

Plasmid	Restriction site inserted	Forward/Reverse	Sequence 5' to 3'
pTM1.W-NiV_M N	NheI	Forward	ATCATTGCTAGCTTCATCATGAGTGATATCTTTGAAGAGGCGGCTAG
pTM1.W-NiV_M N	StuI	Reverse	TCATCAAGGCCTCACACATCAGCTCTGACGAAATCAAGGTC
pTM1.W-NiV_M L	NotI	Forward	ATTAAAGCGGCCGCTATTCATCATGGCCGATGAATTATCAATATCCGAC
pTM1.W-NiV_M L	XmaI	Reverse	TAGTAACCCGGGTCAGATAATAGATATGTATCCGATTATCTTCCACC
